# The Effect of Prognostic Nutritional Index in Predicting Clinical Outcomes in Valve Replacement Patients

**DOI:** 10.21470/1678-9741-2023-0503

**Published:** 2025-03-13

**Authors:** Rifat Özmen, Funda İpekten, Gülden Sarı, Aydın Tunçay, Okan Özocak, Fatma Sena Topçu, Ahmet Öztürk, Kürşat Gündoğan

**Affiliations:** 1 Department of Cardiovascular Surgery, Faculty of Medicine, Erciyes University, Kayseri, Turkey; 2 Department of Biostatistics, Faculty of Medicine, Adıyaman University, Adıyaman, Turkey; 3 Department of Biostatistics, Faculty of Medicine, Erciyes University, Kayseri, Turkey; 4 Department of Internal Medicine, Division of Intensive Care Medicine, Faculty of Medicine, Erciyes University, Kayseri, Turkey; 5 Department of Clinical Nutrition, Health Sciences Institute, Erciyes University, Kayseri, Turkey

**Keywords:** Atrial Fibrillation, Nutritional Assessment, Hospital Mortality, Cardiopulmonary Bypass, Aortic Valve, Constriction, Inflammation, Intensive Care Units

## Abstract

**Introduction:**

Cardiopulmonary bypass is known to be a cause of systemic inflammatory response. The systemic inflammatory response affects albumin and lymphocyte levels and is associated with the development of complications. Serum albumin and lymphocyte concentrations have been used to create inflammation-based risk scores, which predict prognosis in different patient groups. One of these risk scores is called the Prognostic Nutritional Index (PNI). In this study, our objective was to examine how changes in PNI values, measured at different times before and after surgery, impact clinical outcomes and hospital mortality.

**Methods:**

One hundred and sixty-four patients were retrospectively scanned and included in the study. Patients were divided into aortic valve replacement (AVR) and mitral valve replacement (MVR) groups. The patient's preoperative and postoperative PNI values were examined. Duration of cross-clamping, cardiopulmonary bypass time, length of hospital and intensive care unit stay, postoperative mortality, atrial fibrillation, and acute kidney injury (AKI) development were evaluated.

**Results:**

Preoperative and second PNI values were lower in the patients that developed AKI and non-survivors. The PNI cutoff value was ≤ 28.01 in non-survivors (*P*=0.001). In the MVR group, the decrease in PNI value over time was statistically significant (*P*<0.001). There was a negative correlation between preoperative PNI value and length of stay in intensive care unit, cross-clamping, and cardiopulmonary bypass duration (*P*<0.05, *P*<0.01).

**Conclusion:**

A correlation was determined between the PNI value and development of postoperative AKI and mortality. PNI value, an easy method to use, can be used in the follow-up of these patients.

## INTRODUCTION

In today's world, heart valve surgery operations are still performed with cardiopulmonary bypass (CPB). These surgeries, performed with CPB, cause the development of a systemic inflammatory response. This condition is also associated with the development of complications^[1,2]^. It is believed that the contact of blood with artificial surfaces during extracorporeal circulation, surgical trauma, ischemia-reperfusion injury, and endotoxemia play a triggering role in the formation of inflammatory reactions^[1,2,3,4]^. Severe clinical outcomes that can result in death can occur in approximately 7.9% of cases associated with increased inflammatory response. Therefore, investigating the role of direct and indirect indicators of inflammatory changes in patient prognosis has become a high priority goal for the development of therapeutic and preventive strategies^[3,4,5]^.

Various scoring systems are used in daily practice to evaluate patients during the preoperative period and to predict potential risks. The Society of Thoracic Surgeons score and the European System for Cardiac Operative Risk Evaluation are used for this purpose^[6]^. However, albumin levels are not included in these scoring system parameters. Various studies in the literature emphasize that the nutritional status is associated with the development of cardiac cachexia, postoperative clinical outcomes, and mortality in various diseases^[6,7]^. Deterioration in various indicators of nutritional status affects survival by causing immune system and wound healing disorders^[8,9]^. Based on this, inflammation-based risk scores that indicate prognosis in various patient populations were created using serum albumin and lymphocyte concentrations. The Prognostic Nutritional İndex (PNI) is one of these and is a biomarker used for this purpose in different disease groups. PNI was first defined in 1980, by Buzby et al.^[10]^, and subsequently updated by Onodera et al.^[11]^.

However, we observed that there is no study examining the relationship between the development of complications and mortality after isolated mitral valve replacement (MVR) in the literature, while there are limited number of studies published in patients who underwent aortic valve replacement (AVR)^[13,14,15,16,17]^. We aimed to investigate whether the change in postoperative PNI values measured at different times, which we hypothesized based on this study, would have an effect on predicting clinical outcomes and hospital mortality in patients undergoing isolated AVR or MVR.

## METHODS

The approval of the local ethics committee of the Faculty of Medicine of Erciyes University was obtained on 08/02/2023 (approval number 2023/109). The data of 399 patients who underwent surgery for heart valve replacement between 01/01/2015 and 12/31/2022 in the Department of Cardiovascular Surgery at the Faculty of Medicine of Erciyes University were retrospectively scanned from the hospital record system.

**Fig. 1:**
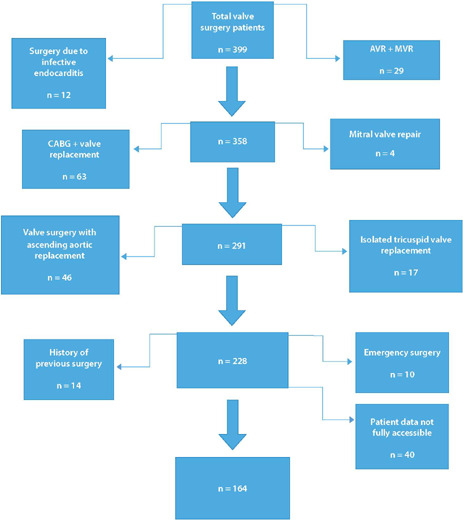
The inclusion criteria scheme. AVR=aortic valve replacement; CABG=coronary artery bypass grafting; MVR=mitral valve replacement.

The patients whose AVR or MVR procedures were recorded were included in our study. Patients who have previously undergone open heart surgery, patients who have undergone simultaneous heart valve replacement with coronary artery bypass grafting (CABG), patients who have undergone aortic aneurysm or aortic dissection resulting in ascending aortic replacement along with valve replacement, patients who have undergone combined valve replacement, patients who have undergone heart valve surgery due to infective endocarditis, patients who have undergone mitral or aortic valve repair, patients for whom complete access to file data was not possible, and patients who were taken into surgery under emergency conditions were excluded from the study. The detailed data regarding the inclusion criteria were summarized in Figure 1. All patients were operated on by the same team and underwent surgery with standard median sternotomy. Arterial cannulation was performed through the ascending aorta. Dual stage venous cannulation was performed in patients undergoing AVR, while bicaval venous cannulation was applied in patients undergoing MVR. Surgical access to the mitral valve was achieved through a left atrial incision in patients undergoing MVR. For AVR, a standard aortotomy incision was used. All patients were cooled down to 32°C. Cardiac arrest was achieved in all patients using cold blood cardioplegia administered in an antegrade fashion.

Data from 164 patients who met the inclusion criteria were retrospectively scanned and included in the study. The body surface area of the patients was calculated. Additionally, the albumin, blood urea nitrogen, creatinine, and complete blood count parameters of the patients were examined in the blood samples collected during preoperative admission, postoperative intensive care admission, and on the first and fifth postoperative days. Detailed data are summarized in the Supplementary Table 1.

**Supplementary Table 1:** 

Variables	Diagnostic group		Z	*P*-value
	Mitral valve replacement group (n=108)	Aortic valve replacement (n=56)		
Preoperative BUN (mg/dL)	19.3 (15.0-24.1)^a,c^	17.2 (14.1-21.8)^a,b^	1.219	0.223
BUN 1 (mg/dL)	17.5 (14.0-21.9)^a^	16.1 (13.2-20.2)^a^	1.397	0.162
BUN 2 (mg/dL)	21.0 (15.8-28.0)^b,c^	19.8 (17.2-24.1)^b^	0.818	0.413
BUN 3 (mg/dL)	22.7 (16.9-33.0)^b^	20.8 (16.9-25.2)^b^	1.774	0.076
*P*-value*	**< 0.001**	**< 0.001**		
Preoperative Cr (mg/dL)	0.9 (0.8-1.1)^a^	0.9 (0.8-1.1)^a^	0.187	0.851
Cr 1 (mg/dL)	0.9 (0.8-1.1)^a^	0.9 (0.8-1.0)^a^	0.918	0.359
Cr 2 (mg/dL)	1.1 (0.9-1.6)^b^	1.1 (0.8-1.4)^b^	1.483	0.138
Cr 3 (mg/dL)	1.1 (0.8-1.6)^c^	0.8 (0.7-1.1)^a^	3.373	**0.001**
*P*-value*	**< 0.001**	**< 0.001**		
Preoperative GFR	75.4 (59.7-98.0)^a^	80.3 (66.4-98.5)^a^	1.206	0.228
GFR 1	78.8 (62.8-99.0)^a^	86.8 (71.3-102.4)^a^	1.852	0.064
GFR 2	58.1 (38.8-81.8)^b^	68.3 (51.3-84.7)^b^	2.106	**0.035**
GFR 3	69.2 (35.4-100.6)^c^	91.2 (64.4-108.2)^a^	3.179	**0.001**
*P*-value*	**< 0.001**	**< 0.001**		
Preoperative AST	23.0 (18.5-33.0)^a^	22.0 (16.0-28.0)^a^	1.226	0.220
AST 1	45.5 (26.8-64.0)^b^	36.0 (28.0-46.0)^b^	2.209	**0.027**
AST 2	67.0 (46.0-105.0)^c^	45.7 (34.1-64.5)^c^	3.439	**0.001**
AST 3	55.0 (33.0-84.0)^a^	33.2 (26.0-50.0)^a^	3.786	**0.000**
*P*-value*	**< 0.001**	**< 0.001**		
Preoperative total protein	7.2 (6.7-7.6)^a^	7.1 (6.8-7.4)^a^	0.194	0.846
Total protein 1	4.9 (4.1-6.8)^b^	4.7 (4.2-5.1)^b^	1.781	0.075
Total protein 2	4.9 (4.5-5.4)^b^	5.1 (4.8-5.5)^c^	1.897	0.058
Total protein 3	5.2 (4.9-5.5)^b^	5.2 (5.0-5.6)^c^	0.992	0.321
*P*-value*	**< 0.001**	**< 0.001**		
Preoperative albumin (g/dL)	4.3 (3.9-4.6)^a^	4.4 (4.3-4.7)^a^	2.074	**0.038**
Albumin 1 (g/dL)	2.9 (2.6-3.8)^b^	2.9 (2.6-3.2)^b^	1.318	0.188
Albumin 2 (g/dL)	3.1 (2.7-3.3)^b^	3.3 (3.0-3.5)^c^	3.086	**0.002**
Albumin 3 (g/dL)	3.2 (3.0-3.4)^b^	3.2 (2.9-3.5)^c^	1.066	0.286
*P*-value*	**< 0.001**	**< 0.001**		
Preoperative WBC (10^3/µL)	7.5 (6.4-8.8)a	7.7 (6.3-9.1)^a^	0.137	0.891
WBC 1 (10^3/µL)	17.0 (12.5-22.3)^b^	14.3 (10.7-18.9)^b^	2.534	**0.011**
WBC 2 (10^3/µL)	15.4 (12.4-18.2)^b^	14.4 (12-18.1)^b^	0.768	0.442
WBC 3 (10^3/µL)	15.6 (12.4-19.9)^b^	14.5 (12.7-17.5)^b^	1.298	0.194
*P*-value*	**< 0.001**	**< 0.001**		
Preoperative Hb (g/dL)	13 (11.3-14.4)^a^	13.9 (12.6-14.9)^a^	2.447	0.014
Hb 1 (g/dL)	9.9 (9.1-10.5)^b^	9.9 (9.2-10.9)^b^	0.341	0.733
Hb 2 (g/dL)	10.0 (9.1-10.8)^b^	10.0 (9.6-10.7)^b^	0.866	0.387
Hb 3 (g/dL)	9.1 (8.7-9.9)^c^	9.2 (8.7-10.1)^b^	0.685	0.493
*P*-value*	**< 0.001**	**< 0.001**		
Platelet_0 (10^3/µL)	239.0 (197.5-291.8)^a^	242.5 (200.0-278.0)^a^	0.068	0.946
Platelet_1 (10^3/µL)	163.0 (122.5-205.5)^b^	166.0 (140.0-213.0)^b^	0.616	0.538
Platelet_2 (10^3/µL)	165.5 (135.3-201.8)^b^	173.5 (134.0-215.3)^b^	0.742	0.458
Platetet_3 (10^3/µL)	132.5 (109.8-173.0)^c^	142.0 (111.0-177.0)^c^	0.786	0.432
*P*-value*	< 0.001	< 0.001		
Preoperative neutrophil (10^3/µL)	4.8 (3.9-6.2)^a^	4.9 (3.6-5.9)^a^	0.742	0.458
Neutrophil 1 (10^3/µL)	14.1 (10.7-18.7)^b^	11.3 (8.9-15.8)^b^	2.701	0.007
Neutrophil 2 (10^3/µL)	13.1 (10.3-15.8)^b^	12.1 (10.2-15.5)^b^	0.660	0.509
Neutrophil 3 (10^3/µL)	13.0 (10.5-18.0)^b^	12.6 (10.6-14.8)^b^	1.049	0.294
*P*-value*	< 0.001	< 0.001		
Preoperative lymphocyte (10^3/µL)	1.7 (1.3-2.2)^a^	2.0 (1.6-2.5)^a^	2.551	**0.011**
Lymphocyte 1 (10^3/µL)	1.5 (1.0-2.7)^a^	1.6 (1.1-2.1)^b^	0.374	0.709
Lymphocyte 2 (10^3/µL)	1.0 (0.7-1.3)^b^	1.0 (0.7-1.1)^c^	0.323	0.747
Lymphocyte 3 (10^3/µL)	1.2 (0.8-1.6)^b^	1.2 (1.0-1.5)^d^	0.174	0.862
*P*-value*	**< 0.001**	**< 0.001**		
Preoperative hematocrit	40.1 (35.5-43.4)^a^	41.2 (38.4-44.8)^a^	2.072	**0.038**
Hematocrit 1	29.9 (27.5-31.8)^b^	29.5 (27.6-32.1)^b^	0.048	0.962
Hematocrit 2	30.3 (27.6-32.6)^b^	30.3 (27.9-31.9)^b^	0.066	0.947
Hematocrit 3	27.5 (25.6-29.4)^c^	27.6 (25.8-29.9)^b^	0.301	0.764
*P*-value*	**< 0.001**	**< 0.001**		

Data are expressed as median (first quartile-third quartile). The same letters in the column indicate intertemporal similarity, and differences indicate difference.

*P-value among the groups

AST=aspartate aminotransferase; BUN=blood urea nitrogen; Cr=creatinine; GFR=glomerular filtration rate; Hb=hemoglobin; WBC=white blood cells

The preoperative PNI value was calculated from the blood values obtained during the patient's hospital stay. Additionally, PNI values were calculated in three consecutive periods to evaluate postoperative changes. The postoperative intensive care admission PNI value of the patients was accepted as the first measurement. The second PNI value measurement was taken on postoperative day one. And the third PNI value measurement was taken on postoperative day five. The PNI value was calculated using the formula: 10 × serum albumin (g/dl) + 0.005 × total lymphocyte count (/mm^3^)^[12]^.

Cross-clamping (XCL) time, CPB times, length of stay (LOS) in the hospital, and LOS in the intensive care unit (ICU) were obtained from the patient’s file. Postoperative mortality, development of atrial fibrillation (AF), stroke, and acute kidney injury (AKI) were evaluated in patients.

The development of postoperative AKI was defined according to the Kidney Disease: Improving Global Outcomes criteria^[12]^. Postoperative atrial fibrillation (POAF) development is defined as patients who have recorded sinus rhythm preoperatively and develop AF during their hospital stay, documented by electrocardiography or treated with electrical or medical cardioversion, and diagnosed as POAF in the patient records. Stroke is described as patients who experience conditions such as weakness, visual impairment, and paralysis during the postoperative period and receive a diagnosis through cranial imaging and neurology consultation.

Mortality is considered surgical-related deaths that occur either in the hospital during surgery or during the post-surgical hospitalization period.

### Statistical Analysis

Histogram and Q-Q plots were examined. Shapiro-Wilk’s test was applied to assess the data normality. Levene’s test was used to test variance homogeneity.

Mann-Whitney U test was used for quantitative variables in comparisons between groups. The Kruskal-Wallis test was used for comparisons between groups of more than two. Comparisons between measures were evaluated with the Friedman test. Pearson χ2 analysis, and Fisher’s exact χ2 test were used to compare categorical data. The Dunn Bonferroni test was applied for multiple comparisons. The relationship between quantitative data was evaluated with Spearman’s correlation analysis. Receiver operating characteristic (ROC) analyses were applied to identify the predictive ability of PNI markers on mortality and postoperative AKI. Cutoff values are determined using Youden index. Using these cutoff values for each marker, sensitivity, specificity, positive, and negative predictive values are calculated with 95% confidence intervals. Analyses were conducted using Turcosa Cloud (Turcosa Ltd Co, Turkey) statistical softwares and R 4.0.0 (www.r-project.org) software. *P*-value < 5% was considered as statistically significant.

## RESULTS

A total of 164 patients were included in the study. Out of these, 108 received MVR and 56 received AVR; 62% of the MVR group were women, while 73% of the AVR group were men. The average age of the patients was 54.35 ± 14.09 years. The median average age in the MVR group was 55.5 (47.3-64.8) years, and in the AVR group, it was 60.5 (44.3-65.8) years. The most common diseases that occurred alongside each other were preoperative AF (29.4%), hypertension (31.7%), and coronary artery disease (31.7%). The average preoperative ejection fraction was 55.46 ± 8.65.

When all patients were evaluated together, the patient's LOS in hospital was 14.49 ± 6.84 days, the LOS in ICU was 4.49 ± 4.5 days. The XCL time was 77.56 ± 25.78 minutes. The CPB time was 135.8 ± 43.03 minutes. And the amount of urine processed by the pump was 1808.06 ± 804.79 mL.

The XCL time in the MVR group was longer compared to the AVR group with 79 (65-90) minutes. There was a statistically significant difference between the groups in terms of XCL time (*P*=0.042). The details of the patients' demographic data and intraoperative variables are summarized in Table 1.

**Table 1:** Patient’s demographic data and intraoperative variables.

Variables	"All patients (n = 164)"	"Mitral valve replacement group (n = 108)"	"Aortic valve replacement group (n = 56)"	χ2	*P*-value
Sex, n (%)					
Female	82 (50.0)	67 (62.0)	15 (26.8)	18.331	**< 0.001**
Male	82 (50.0)	41 (38.0)	41 (73.2)		
Age, years (range)	57.0 (47.0-65.0)	55.5 (47.3-64.8)	60.5 (44.3-65.8)	0.321	0.748
Comorbidities, n (%)					
Preoperative AF	48 (29.4)	47 (43.9)	1 (1.8)	31.420	**< 0.001**
HT	52 (31.7)	39 (36.1)	13 (23.2)	2.833	0.092
CAD	52 (31.7)	35 (32.4)	17 (30.4)	0.072	0.789
DM	26 (15.9)	20 (18.5)	6 (10.7)	1.684	0.194
Thyroid dysfunction	22 (13.4)	16 (14.8)	6 (10.7)	0.534	0.465
CRF	16 (9.8)	15 (13.9)	1 (1.8)	6.136	**0.013**
Stroke	13 (7.9)	11 (10.2)	2 (3.6)	2.210	0.222
COPD	14 (8.5)	9 (8.4)	5 (8.9)	0.013	0.999
BSA (kg\m^2^)	1.8 (1.7-1.9)	1.8 (1.7-1.9)	1.9 (1.7-2.0)	2.628	**0.009**
Carotid artery stenosis					
No stenosis	158 (96.3)	107 (99.1)	51 (91.1)	6.849	**0.018**
< 50 % stenosis	1 (0.6)	0 (0.0)	1 (1.8)		
> 50 % stenosis	5 (3.0)	1 (0.9)	4 (7.1)		
Preoperative EF (%)	57.0 (51.0-60.0)	56.0 (52.0-60.0)	57.0 (50.0-63.0)	0.434	0.665
Length of hospital stay (days)	13.0 (9.0-19.0)	13.0 (8.0-18.0)	14.0 (10.0-20.0)	0.861	0.389
Length of ICU stay (days)	3.0 (2.0-4.8)	3.0 (2.0-5.0)	3.0 (3.0-4.0)	0.066	0.948
XCL duration (minutes)	75.0 (60.0-90.0)	79.0 (65.0-90.0)	70.0 (60.0-85.0)	2.029	**0.042**
CPB duration (minutes)	120.0 (110.0-160.0)	130.0 (110.0-169.3)	120.0 (101.0-140.0)	1.836	0.066
Urine output in CPB (ml)	1700.0 (1100.0-2500.0)	1650.0 (1125.0-2500.0)	1800.0 (1000.0-2275.0)	0.465	0.642

Data are expressed as mean ± standard deviation and n (%)

AF=atrial fibrillation; BSA=body surface area; CAD=coronary artery disease; COPD=chronic obstructive pulmonary disease; CPB=cardiopulmonary bypass; CRF=chronic renal failure; DM=diabetes mellitus; EF=ejection fraction; HT=hypertension; ICU=intensive care unit; XCL=cross-clamping

POAF was developed in 40 patients (24.5%). AKI was developed in 40 patients (24.4%). Stroke was developed in two patients (1.2%). Deep surgical site infection was developed in one patient (0.6%). In-hospital mortality occurred in 26 patients (15.9%). There was no statistically significant difference between the MVR and AVR groups in terms of postoperative AKI, stroke, POAF, deep surgical site infection, and in-hospital mortality. The clinical outcomes of the patients are summarized in Table 2.

**Table 2:** Postoperative clinical results of patients according to diagnosis groups.

Variables	Patient group			χ2	*P*-value
	All valve replacement patients (n = 164)	Mitral valve replacement group (n = 108)	Aortic valve replacement group (n = 56)		
AF (%)	40 (24.5)	25 (23.4)	15 (26.8)	0.232	0.630
AKI (%)	40 (24.4)	31 (28.7)	9 (16.1)	3.191	0.074
Stroke	2 (1.2)	2 (1.9)	0 (0.0)	1.050	0.548
Deep surgical site infection	1 (0.6)	0 (0.0)	1 (1.8)	1.940	0.341
Hospital mortality	26 (15.9)	20 (18.5)	6 (10.7)	1.684	0.194

Data are expressed as n (%)

AF=atrial fibrillation; AKI=acute kidney injury

**Fig. 2:**
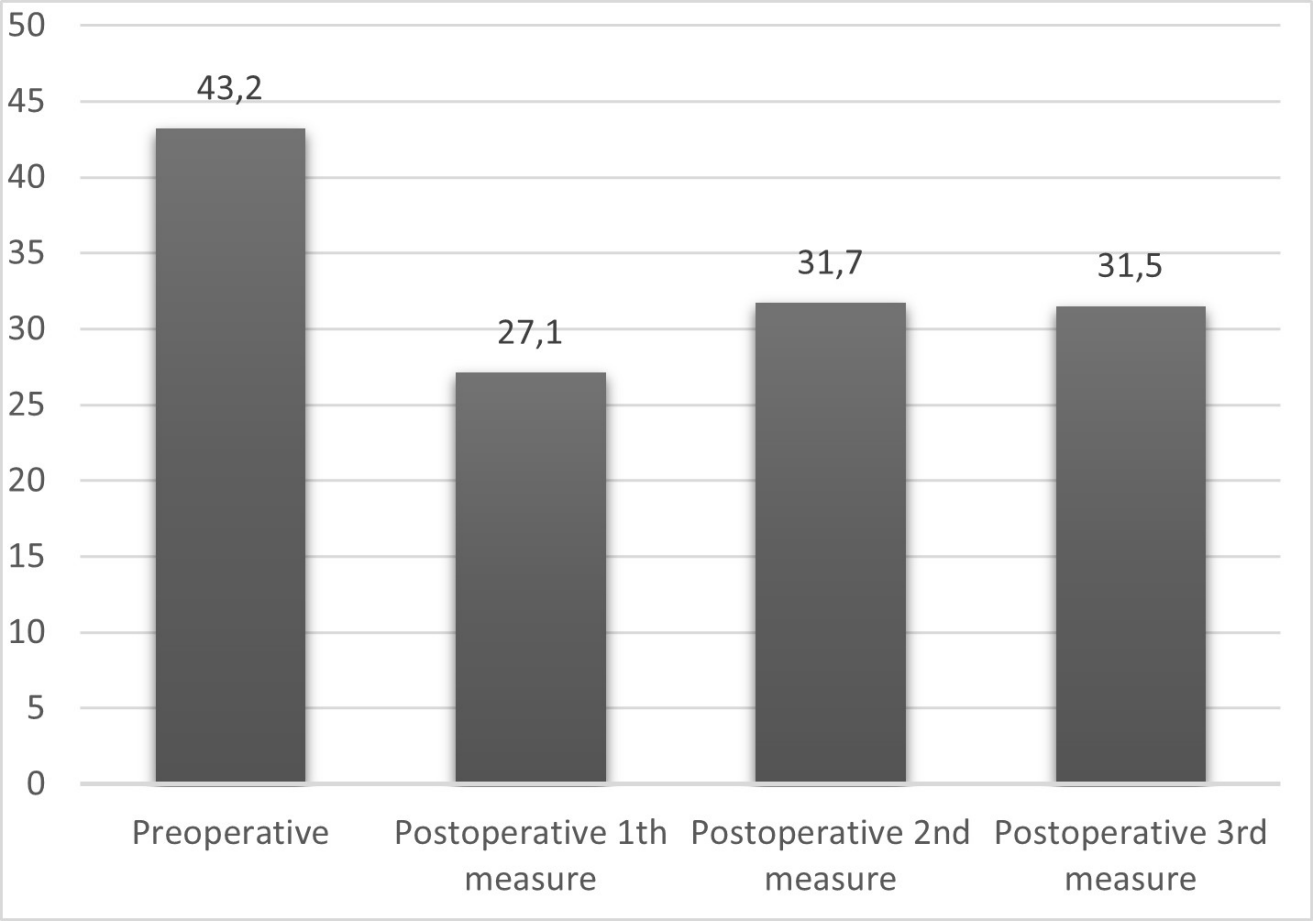
Prognostic Nutritional Index values in mitral valve replacement patients.

**Fig. 3:**
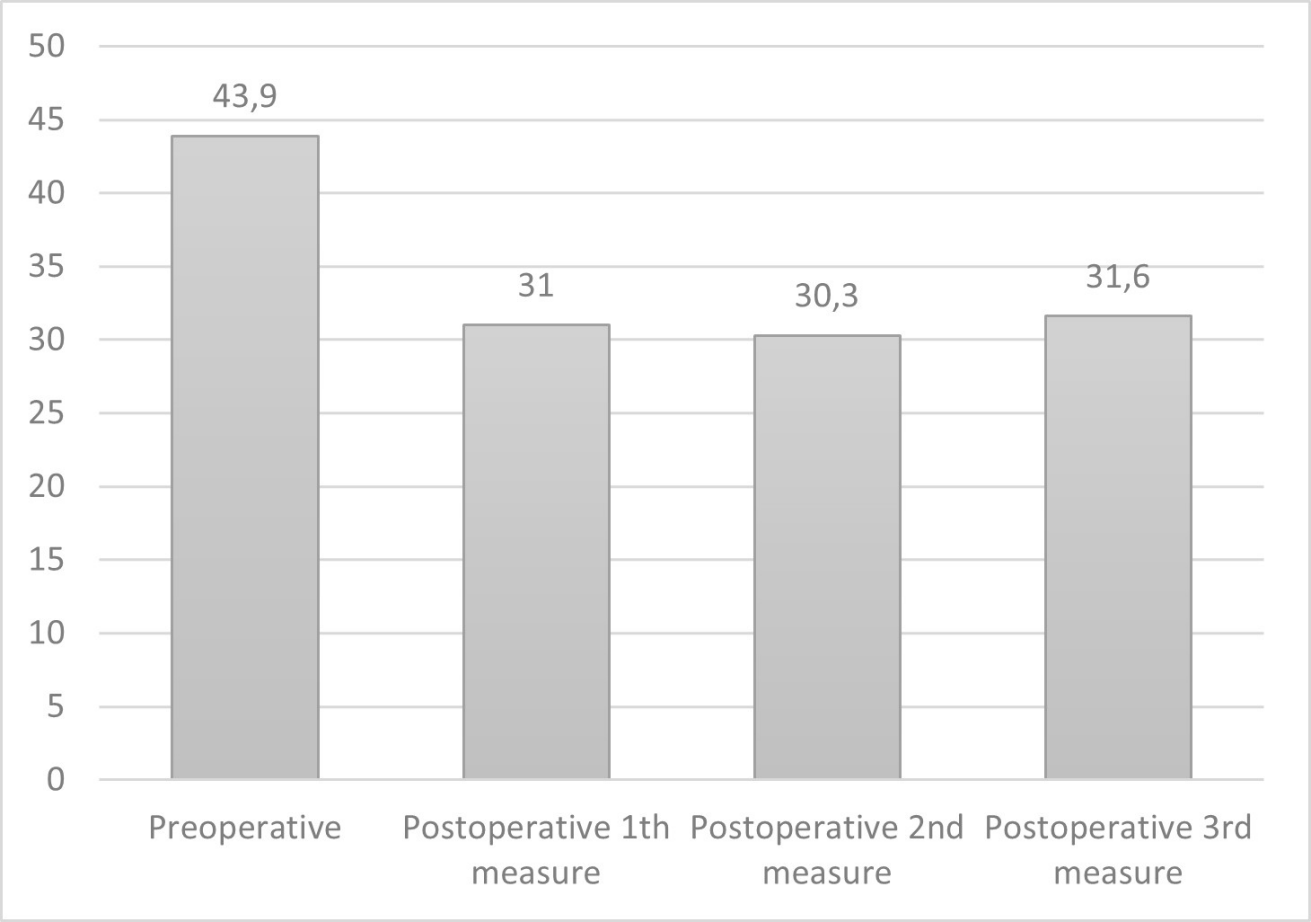
Prognostic Nutritional Index values in aortic valve replacement patients.

The preoperative PNI value was calculated as 43.2 (38.9-45.9) in the MVR group and 43.9 (38.4-46.8) in the AVR group. A statistically significant difference was found between the groups in terms of preoperative PNI value (*P*=0.038). When the changes of PNI according to time were evaluated by groups, it was determined that there was a decreasing trend in PNI in both MVR and AVR groups compared to the preoperative value in each consecutive three measurements (Figure 2 and 3).

When comparing the MVR and AVR groups, it was observed that there was a greater decrease in the second measurement value of PNI in the MVR group, and it was determined that this difference was statistically significant (*P*=0.003) (Table 3). When compared with the preoperative value of PNI in both the MVR and AVR groups, it was determined that the decrease in postoperative different three period values was statistically significant according to time (*P*<0.001) (Figure 2 and 3).

**Table 3:** PNI change according to diagnosis groups.

PNI values	Mitral valve replacement group	Aortic valve replacement group	Z	*P*-value
Preoperative value	43.2 (38.9-45.9)^a^	43.9 (42.5-46.8)^a^	2.079	**0.038**
1^st^ value	29.4 (25.9-38.4)^b^	28.4 (25.5-32.0)^b^	1.486	0.137
2^nd^ value	30.8 (27.4-33.2)^b^	32.8 (29.9-35.1)^c^	2.998	**0.003**
3^rd^ value	31.5 (29.5-33.5)^b^	32.0 (29.5-35.1)^c^	1.131	0.258
*P*-value*	**< 0.001**	**< 0.001**		

Data are expressed as median (1^st^ quartile-3^rd^ quartile)

PNI = Prognostic Nutritional Index

The same letters in the column indicate intertemporal similarity, and differences indicate difference

*P-value among the groups

It was determined that preoperative PNI values were lower in the groups that developed postoperative AKI and mortality, and this difference was statistically significant compared to the group that did not develop AKI and mortality (*P*=0.031, *P*=0.022).

No statistically significant difference was found between the development of POAF and PNI values.

When compared with preoperative PNI values, it was determined that there was a statistically significant difference in the time-dependent decrease of postoperative PNI values in patients who developed postoperative AKI, POAF, and mortality (*P*<0.001). The relationship between postoperative AKI, POAF, and mortality development with PNI values is summarized in Table 4.

**Table 4:** Relationship between postoperative clinical outcomes and PNI.

PNI values	Postoperative AKI development		z	P-value	Postoperative AF development		z	P-value	Mortality		z	P-value
	Developed (n=39)	Non-developed (n=125)			Developed (n=26)	Non-developed (n=138)			Developed (n=25)	Non- developed (n=82)		
Preoperative value	42.2 (36.5-44.3)^a^	43.8 (39.9-46.5)^a^	2.157	**0.031**	42.5 (38.0-45.0)^3^	43.8 (39.8-46.2)^a^	1.113	0.266	42.5 (36.9-44.2)^a^	43.9 (41.0-46.4)^a^	2.298	**0.022**
1^st^ value	27.9 (24.0-38.7)^b^	30.2 (26.0-38.4)^b^	1.229	0.219	26.0 (23.4-39.9)^b^	30.0 (26.2-38.5)^b^	1.440	0.150	26.4 (23.8-37.0)^b^	29.3 (25.9-34.4)^b^	1.263	0.207
2^nd^ value	28.3 (25.5-31.3)^b^	31.7 (28.5-33.7)^b^	3.237	**0.001**	30.5 (26.4-32.6)^b^	31.1 (28.0-33.8)^b^	1.254	0.210	27.8 (25.5-33.2)^b^	31.6(28.6-34.0)^c^	2.220	**0.026**
3^rd^ value	31.4 (29.1-34.3)^b^	31.6(29.9-33.0)^b^	0.212	0.832	31.0 (28.1-32.5)^b^	31.8(29.9-33.9)^b^	1.522	0.128	30.8 (28.1-32.3)^b^	31.8(29.6-34.0)^c^	1.630	0.103
*P- *value*	**< 0.001**	< **0.001**			< **0.001**	< **0.001**			< **0.001**	**< 0.001**		

Data are expressed as median (1st quartile-3rd quartile)

The same letters in the column indicate intertemporal similarity, and differences indicate difference

AF=atrial fibrillation; AKI=acute kidney injury; PNI=Prognostic Nutritional Index

*P-value among the groups

When all the valve patients were evaluated together, a negative correlation was found between preoperative PNI value and ICU stay time (*P*<0.05). Additionally, it was determined that there was a negative correlation between the second measurement value of PNI and XCL and CPB durations (*P*<0.01). It was determined that there is a negative correlation between PNI values and ICU stay time in both the AVR and MVR groups (*P*<0.05). Similarly, a negative correlation was found between PNI values and XCL and CPB times (*P*<0.05 and *P*<0.01). The correlation findings between patients' PNI values are summarized in Table 5.

**Table 5:** Results of correlations among variables.

Variables	Preoperative value	1^st^ value	2^nd^ value	3^rd^ value
**AVR patients**				
LOS in hospital	-0.152	-0.175	-0.194	-0.112
Length of ICU stay	-0.086	-0.336*	-0.216	-0.055
XCL time	-0.067	-0.086	-0.144	0.132
CPB time	0.106	-0.229	-0.108	0.199
**MVR patients**				
LOS in hospital	-0.057	-0.058	-0.083	0.097
Length of ICU stay	-0.220*	-0.029	-0.169	-0.152
XCL time	-0.043	-0.006	-0.231*	-0.153
CPB time	-0.160	0.077	-0.352**	0.003
**All patients**				
LOS in hospital	-0.075	-0.089	-0.098	-0.039
Length of ICU stay	-0.178*	-0.091	-0.157	-0.099
XCL time	-0.066	-0.003	-0.236**	-0.060
CPB time	-0.100	0.009	-0.303**	0.051

*P<0.05, **P<0.01

AVR=aortic valve replacement; CPB=cardiopulmonary bypass; ICU=intensive care unit; LOS=length of stay; XCL=cross-clamping

## DISCUSSION

It is reported that approximately 10-25% of patients who undergo cardiac surgery and experience surgical-related adverse events during the postoperative period have an increase in inflammatory response and changes in albumin levels. Various indexes including albumin values, such as PNI, geriatric nutritional index, and controlling nutritional status score, are used during the preoperative evaluation process in patients undergoing open-heart surgery for this purpose^[13,14,15,16]^. Gürbak et al.^[17]^ reported that a low PNI score is a predictor of all-cause mortality in patients with severe aortic stenosis who undergo surgical AVR (SAVR) treatment. They also stated that the parameter most affected by malnutrition and cachexia that develops with advanced age is the serum albumin level. In the same study, it is emphasized that the mortality rate due to all causes after SAVR is higher in patients with low albumin levels^[17]^. It has been understood that PNI score, which has a better predictive value than albumin levels alone, has started to be used in the follow-up of patients with gastrointestinal malignancies. Over time, it has also been reported in the literature that it might be a prognostic predictor for many chronic diseases, primarily cardiovascular diseases^[10,16,18,19]^. Lee et al.^[15]^ reported that in patients with a PNI score of ≤ 46.1 who undergo cardiac surgery, there is a significant increase in the development rate of complications, including morbidity, mortality, and prolonged stay in the ICU, during the early period. Okuno et al.^[13]^ reports that low PNI levels are an important predictor of mortality in patients undergoing transcatheter AVR^[14]^. Gürbak et al.^[17]^ reported that the PNI value is significantly and strongly correlated with overall mortality in patients with a PNI value ≤ 49.2. They also stated that there was no statistically significant relationship between PNI score > 49.2 and in-hospital mortality being higher. Gurbak et al.^[17]^ reported that the PNI value is significantly and strongly associated with all-cause mortality in patients with a PNI value ≤ 49.2. The same study reports that in patients with a PNI score > 49.2, higher in-hospital mortality was observed, although they did not find a statistically significant relationship.

Kahraman et al.^[14]^ emphasize that hospital mortality is higher in patients with infective endocarditis and whose PNI level is < 35.6.

In our study, mortality was observed in patients with a PNİ value ≤ 28.01. We found a statistically significant relationship between low PNI levels and mortality development, hospital mortality occurred in patients with lower preoperative PNI and second PNI values. When we examined the literature, we observed that other researchers only conducted a single PNI measurement. Differently from the literature, due to both preoperative and postoperative measurements that we conducted, we found a lower PNI cutoff value.

As we have mentioned before, the parameters used to calculate PNI are serum albumin value and lymphocyte count. Albumin, synthesized by hepatocytes, is the most abundant protein in plasma. In addition to its role in determining osmotic pressure, it is also a good indicator of nutritional status and has antioxidant and anti-inflammatory properties^[14,20,21]^. Hypoalbuminemia, which emerges as a consequence of chronic inflammation, is a strong biomarker in predicting mortality and development of AKI in cardiovascular system diseases. It is reported that complications such as heart failure, kidney failure, and septic shock are more frequently observed in patients with infective endocarditis who have a lower PNI value^[14]^. Furthermore, it is emphasized in the literature that lymphopenia and hypoalbuminemia are independent risk factors for AKI^[21,22,23]^. A low PNI value primarily leads to a decrease in intravascular osmotic pressure created by albumin. This condition can indirectly reflect a poor nutritional status, which could potentially contribute to an increase in AKI. Dolapoglu et al.^[12]^ report that there is a statistically significant relationship between a lower PNI value and the development of AKI in patients who have undergone CABG. In our study, we found that the mortality rate is higher in patients with a cutoff albumin value < 2.8 through ROC analysis. In addition to the information in the literature, we have observed that the PNI value in the group with mortality tends to decrease over time in postoperative measurements. We have determined that our finding is associated with mortality development and statistically significant. Our findings regarding the relationship between PNI and mortality development are in line with the literature.

In our study, we found that the preoperative PNI value was lower in the postoperative AKI developed group. Additionally, we observed a decreasing trend in all three postoperative measurement values over time in patients who developed postoperative AKI. We determined that this decrease was statistically significant. Our findings were consistent with the literature. The relationship between AKI development and hypoalbuminemia was found to be similar to the literature in our study^[14,21,22]^. We observed a negative correlation between PNI value and ICU stay duration. We found that patients with a low preoperative PNI level stayed longer in the ICU. Our finding is supported by Lee et al.^[15]^ in the literature.

The pathogenesis of AKI after cardiac surgery is multifactorial. The release of proinflammatory agents, hemodynamic changes during CPB, and predisposing factors specific to the patient can trigger AKI^[24]^. The kidneys are sensitive to hemodynamic changes that occur during cardiac surgery. The activated complement system, up-regulation of cytokines, activation of platelets, free oxygen radicals, leukocyte migration, and excessive fluid overload in the renal cellular interstitium are highlighted causes. As a result, cytotoxic tubular injury occurs. It has been reported in the literature that CPB can also cause these conditions^[1,2,3,4,5,12]^. The renal medulla is more susceptible to ischemia compared to other organs due to its unique circulation characterized by limited reserve and low oxygen tension. Its development is directly related to the duration of CPB and XCL times^[1,2,3,4,5,12]^. In our study, a negative correlation was observed between XCL, CPB durations, and PNI. This negative correlation was found to be statistically significant in the second postoperative measurement. These correlations and findings are supported by the literature.

POAF is one of the most common complications encountered following cardiac surgery. It both increases treatment costs by prolonging the duration of stay in the ICU and leads to an increase in mortality and morbidity. In literature, although there have been reports of a relationship between low albumin levels and POAF in different publications, there are also authors who have reported not finding such a relationship^[25]^. In our study, a significant statistical relationship between low albumin levels and PNI levels could not be determined via POAF.

### Limitations

One of our work's imitations is to be designed retrospectively and to have a relatively low number of patients. Additionally, the lack of preoperative and postoperative C-reactive protein values in our patients, as well as the absence of measurement of nutritional parameters and information on nutrition, constitute other limitation factors.

## CONCLUSION

The conclusion of this study revealed that there is a correlation between the PNI value, which is an indicator of inflammation, and the development of postoperative AKI and mortality. It was also identified that there is a greater decrease in the second measurement value of PNI in the MVR group.

## Abbreviations, Acronyms & Symbols

AF: Atrial fibrillation
AKI: Acute kidney injury
AST: Aspartate aminotransferase
AVR: Aortic valve replacement
BSA: Body surface area
BUN: Blood urea nitrogen
CABG: Coronary artery bypass grafting
CAD: Coronary artery disease
COPD: Chronic obstructive pulmonary disease
CPB: Cardiopulmonary bypass
Cr: Creatinine
CRF: Chronic renal failure
DM: Diabetes mellitus
EF: Ejection fraction
GFR: Glomerular filtration rate
Hb: Hemoglobin
HT: Hypertension
ICU: Intensive care unit
LOS: Lenght of stay
MVR: Mitral valve replacement
PNI: Prognostic Nutritional İndex
POAF: Postoperative atrial fibrillation
ROC: Receiver operating characteristic
SAVR: Surgical aortic valve replacement
WBC: White blood cells
XCL: Cross-clamping
